# Host Genetic Variants in the Pathogenesis of Hepatitis C

**DOI:** 10.3390/v4123281

**Published:** 2012-11-22

**Authors:** Monika Rau, Katharina Baur, Andreas Geier

**Affiliations:** 1 Division of Hepatology, Department of Internal Medicine II, University Hospital Würzburg, Oberdürrbacherstraße 6, 97080 Würzburg, Germany; Email: rau_m@medizin.uni-wuerzburg.de; 2 Department of Gastroenterology and Hepatology, University Hospital Zurich, Rämistrasse 100, 8091 Zurich, Switzerland; Email: kathrin_baur@hotmail.com

**Keywords:** host genetics, Hepatitis C infection, IL28B, SVR, fibrosis progression

## Abstract

Direct-acting antiviral drugs (DAAs) are currently replacing antiviral therapy for Hepatitis C infection. Treatment related side effects are even worse and the emergence of resistant viruses must be avoided because of the direct-antiviral action. Altogether it remains a challenge to take treatment decisions in a clinical setting with cost restrictions. Genetic host factors are hereby essential to implement an individualized treatment concept. In recent years results on different genetic variants have been published with a strong association with therapy response, fibrosis and treatment-related side effects. Polymorphisms of the *IL28B* gene were identified as accurate predictors for therapy response and spontaneous clearance of HCV infection and are already used for diagnostic decisions. For RBV-induced side effects, such as hemolytic anemia, associations to genetic variants of inosine triphosphatase (ITPA) were described and different *SLC28* transporters for RBV-uptake have been successfully analyzed. Fibrosis progression has been associated with variants of Vitamin D receptor (VDR) and *ABCB11* (bile salt export pump). Cirrhotic patients especially have a high treatment risk and low therapy response, so that personalized antiviral treatment is mandatory. This review focuses on different host genetic variants in the pathogenesis of Hepatitis C at the beginning of a new area of treatment.

## 1. Introduction

About 150 million people are infected by the Hepatitis C virus (HCV). The majority of patients have chronic infection and only 10%–20% clear the virus spontaneously [1]. Chronic Hepatitis C infection progresses to fibrosis and in 20%–30% of cases leads to liver cirrhosis with the risk of hepatocellular carcinoma (HCC) (4%) [2]. In the western world the most common cause for liver transplantation is HCV related end stage liver disease [3] and every year 350,000 patients die from Hepatitis C related liver diseases. Antiviral therapy is currently changing and various direct-acting antiviral drugs have been developed and tested in clinical trials. Patients requiring antiviral treatment are heterogeneous and include inexperienced as well as experienced patients such as relapsers, partial-responders or non-responders to former antiviral combination therapy with pegylated-Interferon (Peg-INF) and ribavirin (RBV). Non response means the viral load decreasing to less than 2 log IU/mL during antiviral treatment. Partial response is a decrease of more than 2 log IU/mL, but still detectable at week 12 and 24, and relapse is the reappearance of HCV RNA after antiviral treatment. Additionally costs of treatment regimens with new drugs are rising. Therefore reliable and efficient treatment predictors are needed to reduce and avoid unnecessary therapy costs. Thus, an individualized therapy concept in clinical practice is mandatory.

This review focuses on the host genetics in patients with chronic Hepatitis C infection described and analyzed in recent years. The first part pictures single nucleotide polymorphisms (SNPs) with strong prediction for antiviral therapy response (Table 1). Secondly, genetic variants are discussed with predictive values for fibrosis progression in these patients. 

## 2. Host Genetics as Predictive Markers for Therapy Response

### 2.1. Interleukin28B

In 2009 three genome-wide association studies (GWAS) found genetic polymorphisms (SNPs) within the *IL28B l* ocus on chromosome 19 determined the outcome of HCV therapy. Ge *et al.* identified rs12979860 (3 kilobases upstream of the *IL28B* gene encoding the type III interferon IFN-l3) in patients with HCV genotype (GT) 1 infections with the strongest association to therapy response. The CC genotype showed a twofold greater rate of sustained virological response (SVR) in Europeans and Hispanics and a threefold higher rate in African-Americans in comparison to Non-CC genotype. Interestingly, CC genotype was not only associated with better treatment response but also with a higher viral load indicating the *IL28B* impact on intrahepatic interferon (INF) regulation genes. Other variants such as rs28416813 and rs8103142 were highly associated to rs12979860 in this study cohort [4]. In 154 Japanese patients with GT 1 another GWAS identified two SNPs rs8099917 and rs12980275 lying between *IL28B* and *IL28A* with association with non-response treatment. Furthermore four supplementary SNPs located in the *IL28B* region showed strong association to therapeutic non‑response in this replication study (rs8105790, rs11881222, rs8099917, rs7248668). After a logistic regression model, minor allele of rs8099917 was found to be the most significant predictor for therapeutic non‑response [5]. 

Suppiah *et al.* confirmed this finding in an Australian and European cohort with GT 1 infection and Peg-INF/RBV treatment. Rs8099917 showed the strongest association with therapy response amongst others such as rs12980275, rs8105790, rs8103142, rs10853727, rs8109886 [6].

**Table 1:** Host genetics associated with therapy response.

Therapy response			
Gene		SNP	Ref
Interleukin 28B	*IL28B*	rs12979860	[4,7,8,9,10,11,12,13,14,15,16,17,18,19,20,21,22,23,24,25,26,27,28,29,30,31,32,33,34,35,36]
		rs8099917	[5,6,8,9,12,13,15,18,30,32,33,37,38,39,40,41,42,43]
		rs28416813	[4]
		rs8103142	[4,6]
		rs12980275	[5,6,18]
		rs8105790	[5,6,18]
		rs11881222	[5]
		rs7248668	[5]
		rs10853727	[6,18]
		rs8109886	[6]
Concentrative nucleoside transporters	*Slc28A2*	rs11854484	[44,45]
	*Slc28A3*	rs10868138	[46]
		rs56350726	[46,45]
Equilibrative nucleoside transporters	*Slc29A1*	rs6932345	[34]
Inosine triphophatase	*ITPA*	rs1127354	[45,46,47,48,49,50]
		rs7270101	[45,46,47]
Vitamin D receptor NR1I1	*VDR*	rs7975232	[51]
		rs1544410	[51]
		rs731236	[51]
	*CYP27B1-1260*	rs10877012	[52,53]
	*GC-globulin*	rs7041	[54]
		rs4588	[54]
Bile salt export pump	*ABCB11*	1331	[55,56]

#### 2.1.1. Geographic and Ethnic Distribution

Interestingly, the frequency of rs12979860 CC genotype varied in different ethnic groups in these studies. East Asians had a higher percentage of CC genotype [4] and in 1,008 individuals from six independent HCV cohorts the CC genotype had a high frequency in North and East Asia, an intermediate frequency in Europe and only minor frequency in Africa [7]. Calvacante *et al.* analyzed rs12979860 and rs8099917 in admixed population. The genetic background was tested by seven genetic ancestry informative markers. Patients with rs12979860 non-CC genotype and a more African genetic background had less chance to reach SVR in comparison to European and Amerindian genetic background. TT-genotype of rs8099917 was also affected by genetic ancestry and a higher African genetic composition was associated with Non-SVR [8]. In a small Korean study, rs12979860 CC and rs8099917 TT seemed to occur more frequently in the Korean population [9].

#### 2.1.2. *IL28B* in Different HCV Genotypes

As the viral genotype is an important treatment predictor Mc Carthy *et al.* analyzed the rs12979860 in GT 2/3. In this study cohort patients with GT 2/3 were more likely to have the good responder genotype, but the study could not fully explain the difference in SVR, as the SNP and the viral genotype were independently associated with therapy response [10]. An Italian study showed an association of *IL28B* polymorphism rs12979860 with treatment response only in GT 2/3 patients who had not achieved RVR in combination therapy with Peg-INF/RBV [11]. In a retrospective study with 719 patients in Japan, treated with either Peg-INF/RBV or interferon monotherapy, rs8099917 was an independent predictive factor in Peg-INF/RBV patients with GT 2b and in interferon monotherapy patients with GT 2a [37]. In another replication study with 2112 Japanese patients with GT 1b/2a infection, rs8099917 and rs12979860 were associated with treatment response in patients with Peg-INF/RBV as well as with interferon monotherapy. Rs8099917 had the strongest association and was also associated with early viral decline [12]. Moghaddam *et al.* analyzed 281 Scandinavian GT3 infected patients with Peg‑INF/RBV. *IL28B* polymorphisms rs12979860 and rs8099917 showed association with RVR but not with SVR [13]. In GT-4 infected patients, studies with patients from different ethnic groups also confirmed an association between *IL28B* rs12979860 and therapy response in these patients [14,57].

#### 2.1.3. Spontaneous Clearance and Viral Kinetics

Another GWAS of a European cohort (n = 1,362) including all HCV genotypes confirmed *IL28B *rs8099917 as a genetic marker for natural and treatment-induced control of HCV infection. Patients with the *IL28B* rs8099917 G risk genotype had higher risk to fail spontaneous viral clearance and develop chronic infection. After stratifying patients in groups according to the viral genotype and host polymorphisms (rs8099917), a strong association was seen between rs8099917 and therapy response in GT 1/4, but not for GT 2/3 and strong linkage disequilibrium with rs12979860 was described [15]. These associations for rs8099917 were confirmed by another Japanese study for GT 1 [38]. To study spontaneous clearance in more detail Tillmann *et al.* analyzed a German cohort of 136 women infected with a single-source HCV genotype 1b while receiving anti-D immunoglobulin prophylaxis. In this cohort with minimal social and ethnic background variations and known time of exposure *IL28B* rs12979860 was associated with spontaneous viral clearance [16]. This result was also confirmed in other studies [7,17]. In a recent Chinese study spontaneous clearance rate in 450 blood donors was associated with four SNPs (rs8099917 with strongest association, rs81057790, rs12980275 and rs10853728), but no association was found with rs129879860 as described in Caucasian cohorts. Furthermore women were more likely to spontaneously clear the virus than men [18]. 

In a mixed cohort with 196 African-American and 205 Caucasian-American a strong association for early viral kinetics (day 0–28) during treatment with Peg-INF and RBV was described for rs12979860 [19]. *IL28B* polymorphisms rs12979860 showed also association with early viral decline, while initiating Peg-INF/RBV therapy in other study cohorts [20,21,22]. 

#### 2.1.4. Interferon Gene Expression

Hepatic gene expression before and during treatment with Peg-INF/RBV was recently analyzed to find host factors for treatment prediction [58,59]. Up-regulation of interferon-stimulated genes (ISG) was associated with poor treatment response. Honda *et al.* showed in 91 patients a strong association between *IL28B* polymorphism and ISG expression. Patients with the minor allele of rs8099917 showed higher expression of hepatic ISG associated with a poor treatment response [39]. In a cohort of 133 patients with GT 1 and GT 2 infection, CC genotype of rs12979860 was associated with higher expression of genes suppressing the antiviral state and lower expression of genes promoting the antiviral state in liver biopsies. The authors discussed an inverse correlation of these genes with *IL28B* polymorphism [23]. Lopez-Rodriguez *et al.* tested 63 SNPs of genes in the interferon-signaling pathway. In a multivariate logistic regression model, *OASL* rs12819210 and *IFIT1* rs304478 were independent predictors for therapy response and even improved predictive value if used together with *IL28B* rs12979860 [24]. In a mathematical model Scott *et al.* showed an association between rs12979860 and increase of infected hepatocyte death rate in the study cohort of 20 GT 1 patients [25]. Not only hepatic gene expression, but also gene expressions of 153 human genes of INF-related pathways were analyzed in PBMCs of 56 patients during antiviral treatment with PEG-INF and RBV [26]. In patients with rs12979860 T/C or TT genotype, pre-activation of ISGs was seen or the sustained activation of ISGs during treatment failed. In contrast patients with CC genotype showed a sustained treatment-induced gene expression [26]. In 79 Japanese patients with liver biopsy *IL28B,* rs8099917 had no association with histological grading and staging, but a lower nuclear staining of STAT1 in hepatocytes before treatment increased the prediction of SVR in GT1-infected patients with TT-genotype [40].

#### 2.1.5. Treatment Duration

In recent years response guided treatment duration has been established for chronic HCV infection. In patients with GT 1 infection the treatment duration was prolonged up to 72 weeks in patients with slow-response to antiviral treatment. Therapy extension is difficult to tolerate for patients with treatment-induced side effects. This issue was analyzed in two cohorts and patients with rs12979860 non-CC genotypes had higher rates of relapses and only patients with T allele (TT/TC) and slow‑response to antiviral treatment benefit from an extended treatment duration of 72 weeks [27,28]. Mangia *et al.* tested *IL28B* variants for response-guided treatment duration with only limited success. Patients achieving rapid virological response (RVR) all had good therapy response regardless of rs12979860 [29]. Early viral dynamics, in a Japanese study, had only a predictive value for therapy response in patients with rs8099917 TT-genotype. Non reaching RVR at week 4 was *per se* a predictive factor for non-SVR in these cohorts regardless of IL28B [41]. Huang *et al.* analyzed rs8099917 to identify GT-1 patients responding to a short treatment of 24 weeks with Peg-INF/RBV. Only in Non-RVR patients was rs8099917 predictive for treatment response. RVR *per se* was the most important predictive factor [42]. Finally, all these studies confirm that RVR is the best predictor for SVR in patients receiving Peg-INF/RBV as it was described several years ago [60].

#### 2.1.6. *IL28B* Genetics in Clinical Practice

To use *IL28B* polymorphisms in clinical practice Fischer *et al.* analyzed the combined determination of four *IL28B* polymorphisms for the best pre-treatment prediction. Rs12979860 and rs8099917 were the best predictors and rs8099917 showed no increase in positive prediction value for patients with rs12979860CC. However, in patients with rs12979860CT, additional genotyping of rs8099917 significantly improved SVR prediction [30]. The study group of the first GWAS published later an intention-to-treat analysis of the same cohort. In a multivariate regression model *IL28B *polymorphism was the best predictor for therapy response between other predictors such as ethnic background, baseline viral load, hepatic fibrosis stage, fasting glucose level, BMI and RBV starting dose (RVR stronger than IL28B) [31]. Kurosaki *et al.* presented a model for the pre-treatment prediction including host and viral genetic factors such as the *IL28B* polymorphism and mutations in the interferon sensitivity determining region (ISDR). In 469 patients with HCV GT 1 infection this model showed high reproducibility and 70% sensitivity as well as 78% specificity for predicting SVR [43]. Halfon *et al.* analyzed 198 patients for rs12979860 as well as rs8099917. Determination of rs12979860 alone had the highest predictive value for treatment response in this study cohort of GT 1 patients. The authors concluded that rs12979860 seemed to be sufficient for clinical decisions [32]. Current guidelines from the European Association of the Study of the Liver (EASL) describe *IL28B* polymorphisms as useful to identify a patient’s likelihood for treatment response, but with only a low predictive value [61]. In contrast, the American Association for the Study of Liver Diseases (AASLD) suggests *IL28B* polymorphism as a robust predictive marker for treatment decision with Peg-INF/RBV or in combination with DDA. Testing is useful if it impacts the treatment decision of either patient or physician. There are not sufficient data for decisions about treatment duration and drug combination [62]. Studies on *IL28B* genotype based therapy shortening are currently ongoing.

The predictive value for *IL28B* polymorphisms was not limited to treatment with Peg-INF/RBV, but could be confirmed also in 81 therapy-naive Japanese receiving triple therapy with telaprevir. Rs12979860 and rs8099917 were both correlated to SVR in univariate analysis and multivariate analyses identified rs8099917 as an independent predictor [33]. Other studies with Peg-INF/RBV and a new DAA showed similar results in abstracts of the first interim analysis [63,64,65,66,67,68,69]. 

At the moment new studies with interferon-free therapy regimens are being conducted. In a dose‑escalation study with 83 patients treated for 13 days with mercitabine plus danoprevir or placebo (INFORM-1 study), *IL28B* rs12979860 CC genotype was associated with better early viral kinetics with higher reduction in viral RNA [70]. Other interferon-free treatment regimens replicated these findings for *IL28B* genotype [71,72].

In summary, *IL28B* genotyping had a predictive value in combination therapy with Peg-INF/RBV as well as in triple or quadruple therapy regimens with the backbone of Peg-INF/RBV. To evaluate the importance of *IL28B* variants in interferon-free regimens further studies are needed.

### 2.2. Ribavirin Transporters

Ribavirin is now and will remain in the near future an integral part of the antiviral combination therapy. The drug is transported by various equilibrative and concentrative nucleoside transporters (ENTs, CNTs) such as ENT1, ENT2, CNT2, and CNT3. D’Avolio *et al.* analyzed genetic variants of *SLC28A2* (CNT2) and in 115 HCV-infected patients rs11854484 (in the coding region) was the best independent predictor for SVR [44].

*SLC28A3* (CNT3) is considered to be another important target in RBV uptake and metabolism. Recently, genetic polymorphisms rs10868138G/rs56350726T have been reported as protective against RBV-induced hemolytic anemia in a population of 169 patients infected by genotype 1, but no association with therapeutic outcomes was found [46]. In 546 East Asian patients with GT 1b infection and treatment with Peg-INF/RBV *SLC29A1* (ENT1), rs6932345 was an independent predictive factor of treatment response, but only with small impact in comparison to *IL28B *rs12979860 [34]. Recently a study with 195 HCV infected patients showed an association between *SLC28A3* rs56350726 and treatment response with Peg-INF/RBV. Furthermore *SLC28A2* rs11854484 was associated with higher dosage- and body weight-adjusted RBV levels [45].

### 2.3. Inosine Triphosphatase (ITPA)

A recent GWAS showed a significant association between hemoglobin (Hb) drop at week 4 under antiviral therapy and *ITPA* deficiency by rs1127354 and rs7270101, two functional variants in the *ITPA* gene [47]. In 474 Japanese patients rs1127354 was confirmed to be a useful predictor of RBV‑induced anemia [48]. In a distinct subgroup of Japanese cohorts an association was also ascribed to therapy response [48,49]. The impact of these two genetic variants was replicated and Thompson *et al.* showed the same effect on Hb drop over the course of therapy. *ITPA *deficiency was associated with lower rates of RBV reduction. However, no association between genetic variants of *ITPA *and treatment outcome was seen in logistic regression [73]. These findings were replicated in other studies [46,45].

For treatment-related thrombocytopenia a recent GWAS found rs11697186 in the *DDRGK1* gene with strong association to reduced platelet counts. Furthermore this SNP had strong linkage disequilibrium with rs1127354 as a functional *ITPA* variant [50]. 

### 2.4. Vitamin D Receptor (VDR)

A recent study analysed genetic variants of VDR in patients with GT 1 infection and Peg-INF/RBV. The bAt [CCA]-haplotype was associated significantly with non-response to antiviral therapy. The contributing ApaI rs7975232 CC genotype was similarly associated with Non-SVR and this relation was independent of *IL28B *genotype. In a combined analysis, 25-OH Vitamin D levels affected non-SVR only in patients with the unfavorable NR1I1 CCA (bAt) haplotype [51]. On first view the observed effects are in contrast to the study of Lange, where the FokI polymorphism of the *VDR* was not significantly associated to SVR [52]. However in the same work the *CYP27B1-1260* promotor polymorphism, which may regulate the 1-alpha-hydroxlation of 25-OH Vitamin D, was associated with chronic Hepatitis C. Accordingly, HCV infected patients with the genotype AA of the 1α-hydroxlase promoter polymorphisms indicated higher serum concentrations of 1,25-dihydroxy Vitamin D than patients with genotype AC and CC. In line they observed that the SVR rate was higher in the group with AA or CA *CYP27B1-1260* genotype [52]. These results were replicated in a larger population with more than 700 HCV infected patients [53]. 

Falleti *et al.* showed recently that genetic variants in the *GC-globulin* gene, the main serum Vitamin D binding protein, and Vitamin D deficiency play a complementary role in predicting antiviral therapy [54]. These association studies are underlined by two recent independent studies that showed direct antiviral effect of Vitamin D in cell lines [35,74]. 

In line with these findings in genetic studies, a significant correlation between low 25-OH Vitamin levels and decreased responsiveness to interferon-based therapy was described in genotype 1 Hepatitis C population and later confirmed in patients ofr HCV genotype 2 and 3 [52,75]. Bitetto *et al.* showed that Vitamin D levels and *IL-28B* rs12979860 polymorphism were two independent predictors of SVR. Patients with Non-CC genotype of the *IL-28B* rs12979860 polymorphism and with Vitamin D deficiency had highest risk for therapy failure. Vitamin D levels were complementary to the predictive value for antiviral treatment [36].

Most importantly, substitution of Vitamin D to Peg-INF/RBV for treatment-naïve patients with chronic HCV genotype 1 and 2/3 infection indeed improved the treatment response [76,77]. Despite these promising results, further studies are needed to finally elucidate the underlying antiviral mechanism of Vitamin D and its role in clinical practice. 

### 2.5. Bile Salt Export Pump (Bsep)

Cholestasis is frequent in end-stage liver disease. In recent years different studies reported an effect of bile acid viral replication *in vitro* [78,79]. Subsequently, in two independent study cohorts increased bile acid serum levels were associated to a non-response to Peg-INF/RBV [80,81].

The canalicular secretion of bile acids in the liver is mediated by the bile salt export pump (BSEP; gene nomenclature *ABCB 11*), a gene target product of the farnesoid-X-recptor (FXR gene nomenclature *NR1H4*). Due to the fact that an inhibition of the Bsep results in a reduced bile salt secretion and therefore leads to elevated bile acid levels, the relation between polymorphisms in *ABCB 11 *gene and antiviral treatment response have been investigated.

In a HCV population association between the *ABCB 11 *1331C allele, which predisposes to a cholestatic phenotype [82], and Non-SVR was found particularly in the subgroup of HCV genotype 2 and 3 infected patients [55]. In the multivariate analysis the odd’s ratio for SVR according to *ABCB 11* 1331 T *vs.* C was 2.94. In the same work patients with an absence of sustained response had significantly elevated bile acids level than those with SVR [55]. These results were replicated in another study cohort of 353 HCV-patients [56].

## 3. Host Genetics and Chronic Complications Such as Fibrosis Progression

Chronic HCV infection leads to fibrosis and lastly cirrhosis with a high risk for HCC development and finally a need for liver transplantation. In recent years, several small studies were published showing association of different genetic variants and fibrosis progression, but mostly these findings were not replicated in independent studies. Genetic variants associated with fibrosis progression and replicated in different studies are listed in Table 2.

**Table 2:** Host genetics associated with fibrosis progression. Genetic variants were replicated in different studies.

Fibrosis progression			
Gene		SNP	Ref
Interleukin 28B	*IL28B*	rs12979860	[83,84,85]
		rs8099917	[83,86,87]
Vitamin D receptor NR1I1	*VDR*	rs7975232	[88]
		rs1544410	[88]
		rs731236	[88]
Bile salt export pump	*ABCB11*	1331	[89]
Patatin-like phospholipase 3	*PNPLA3*	rs738490	[90,91]
Ring finger protein 7	*RNF7*	rs16851720	[92]
Receptor tyrosine kinase MerTK	*MERTK*	rs4374383	[92]
Tubby like protein 1	*TULP1*	rs9380516	[92]

### 3.1. IL28B

To study the course of chronic infection in the context of genetic variants 364 patients with liver biopsies were genotyped for *IL28B* rs8099917. Positive association was reported for histological proven liver fibrosis and wild—type HCV core amino acids 70 and 91 were significantly more common in rs8099917 T allele. IL28B allele was independently associated with fibrosis and inflammatory activity and also correlated to serum gamma-GPT levels [86].

However, in 247 Italian patients no association between *IL28B* rs12979860 and rs8099917 and fibrosis progression was observed [83].

Interestingly, in a Caucasian population rs8099917 non-TT genotype carriers with Non-1 genotype infection showed slow fibrosis progression as well as reduced necroinflammatory activity in liver biopsies [87]. *IL28B * rs12979860 TT-genotype was in 629 Italian patients an independent predictor of a higher Ishak staging score [84]. Fabris *et al.* analyzed retrospectively in a longitudinal study the fibrosis progression in Hepatitis C GT 1–4 patients. Over a period of 10 years Non-CC carriers with a serum cholesterol ≤175 mg/dL had fibrosis progression more frequently. Both factors were independently predictors for fibrosis progression [85].

Nevertheless, to determine the role of *IL28B* in fibrosis progression for clinical decision making further studies are needed.

### 3.2. VDR

In the liver hepatic stellate cells, as the crucial cell type of fibrogenesis express both VDR mRNA and functionally active protein. In contrast hepatocytes are characterized by low levels of VDR mRNA and protein. Recently the effect of the Vitamin D receptor signaling on the fibrogenesis was explored in a Hepatitis C infected population. Genetic variants in the VDR gene were identified as a predisposition for an accelerated fibrosis progression rate in patients with chronic Hepatitis C. For this purpose the reviewed HCV group was divided into rapid (>0.101 U/year) and slow fibrosers (<0.101 U/year). The common bAt [CCA]-haplotype and the contributing ApaI rs7975232 CC genotype of the VDR gene were both significantly associated with patients with an accelerated fibrosis progression rate. In addition to this the same haplotype and same genotype were associated with the presence of cirrhosis. [88]. In accordance to the known anti-inflammatory effect of the VDR signaling, elevated ALT levels were measured in HCV infected patients with the bAt [CCA]-haplotype and the contributing ApaI rs7975232 CC genotype [88]. 

MRNA expression of matrix metalloproteinase 9 (MMP 9), a fibrogenesis activator, was accelerated in chronic HCV infection [93] and correlated to VDR genetic variants [88]. In a HIV-HCV co-infected cohort of 189 patients, low serum 25(OH) D3 levels correlated significantly with severity of liver fibrosis, but were not associated with therapy response or severity of immunodeficiency [94]. 

All these findings fuel the speculation that variants in the Vitamin D receptor gene accelerate the progression to fibrosis and modify the clinical course of chronic Hepatitis C infection.

### 3.3. Bsep

In pediatric cholestatic liver disease hepatocellular accumulation of bile acids induced an up‑regulation of monocytes chemotaxis protein-1, which is involved in early hepatic fibrogenesis [95]. In this context Iwata *et al.* analyzed whether there is an association between *ABCB11* or NR1H4 (*FXR*, the gene activator of BSEP) polymorphisms and fibrosis progression [89].The “cholestatic” *ABCB11* 1331C allele and corresponding CC genotype was associated with the presence of cirrhosis in a HCV‑infected study population. In the multivariate analysis with known risk factors such as age, gender, BMI, HCV genotype and disease duration the association of the *ABCB11* 1331CC genotype with cirrhosis was highly significant with an OR of 5.10. Based on the low minor allelic frequency of the *NR1H4*-1G>T polymorphism in HCV infected patients no association between the distribution of the genotype in relation to the fibrosis stage was observed [89]. 

In a study of 649 HCV-infected patients and 413 controls, risk allele C carriers were overrepresented, but liver stiffness was not associated according to *ABCB11* 1331 genetic variants [96]. No significant association between *ABCB11 *polymorphism and prevalence of cirrhosis either in patients with nonalcoholic fatty liver disease (NASH) [89] or in a population with alcoholic liver disease [97] could be identified. This leads to the speculation that the association of *ABCB11* 1331 genetic variants to fibrosis progression could be HCV-specific.

### 3.4. Different Genes

Falleti *et al.* tested different genetic variants within the *Interleukin-6* gene and observed fibrosis progression in 121 patients with normal transaminases suggesting low inflammatory activity. Grading progression in liver histology was associated with one specific genetic polymorphism of *Interleukin 6*-174G>C [98]. 

Serum levels of YKL-40 as a fibroblast growth factor was evaluated as a non-invasive marker for liver fibrosis in chronic Hepatitis C infection. However, an association between a *YKL-40* promoter polymorphism did not show an association with disease progression in 456 patients of the Hepatitis C Antiviral Long-term Treatment against Cirrhosis Trial (HALT-C) [99].

Many other studies with only small numbers of HCV patients showed association between fibrosis progression and genetic polymorphisms of different genes such as interferon-y receptor, tumour necrosis factor alpha promoter, chemokine receptor 5, RANTES, MCP-2, Interleukin-10, complement factor 5, chemokine scavenger receptor D6, mannan-binding lectin 2, low-density lipoprotein receptor, Factor V Leiden, Factor VII-activating protease antizyme inhibitor (AzI), human platelets antigen (HPA), apolipoprotein E and epidermal growth factor (EGF) [100,101,102,103,104,105,106,107,108,109,110,111,112,113,114]. The list of different genes is not complete and there is a lack of further replication and validation studies with larger numbers of patients. 

A genetic polymorphism in patatin-like phospholipase 3 (*PNPLA3*) associated with steatosis in patients with NASH was also studied in patients with chronic Hepatitis C. In two independent cohorts of chronic Hepatitis C patients, *PNPLA3 *rs738490 GG genotype was independently associated with increased risk of cirrhosis as well as lower treatment response to Peg-INF/RBV and HCC occurrence [90]. In an independent cohort of 537 Caucasian HCV-infected patients *PNPLA3* rs738490 was also associated with steatosis, fibrosis, and fibrosis progression [91]. 

Huang *et al.* tested a cirrhosis risk score (CRS) including seven different SNPs (*AP3S2*, *AQP2*, *AZIN1*, *DEGS1*, *STXBP5L*, *TLR4 and TRPM5*) in 574 patients. This score is associated with higher fibrosis and was replicated in other study populations [115,116,117,118]. It was also assessed in a large study cohort of 938 treatment-experienced patients of the (HALT-C) study population. CRS7 was associated with fibrosis progression and cirrhosis, but not to HCC recurrence and clinical outcome [119]. Most importantly, a recent GWAS with 1161 European patients identified three SNPs of genes regulating apoptosis as relevant for fibrosis progression in chronic Hepatitis C. The first rs16851720 is located in a RNF7 gene encoding an antioxidant that protects for apoptosis. The other two SNPs (*MERTK* rs4374383, *TULP1 *rs9380516) encode for proteins involved in phagocytosis of apoptotic cells [92]. Although these data indicate a functional role of apoptosis genes in fibrosis progression, further studies are needed to replicate these findings. 

## 4. Conclusions

In the area of new direct acting antiviral drugs, host genetics factors contribute to personalize treatment strategies and determine the best treatment strategies for each patient profile. IL28B polymorphisms are already established in clinical practice and facilitate treatment decisions. Further genetic markers need to be replicated and tested for decision making before using in clinics. Furthermore, reliable prediction of therapy success and disease progression will strengthen the patient’s motivation and compliance and thereby even further contribute to a more favorable course of the disease.
